# Varying Collimation for Dark-Field Extraction

**DOI:** 10.1155/2009/847537

**Published:** 2010-02-16

**Authors:** Ge Wang, Wenxiang Cong, Haiou Shen, Yu Zou

**Affiliations:** ^1^SBES Division/ICTAS Center for Biomedical Imaging, VT-WFU School of Biomedical Engineering, Virginia Tech, Blacksburg, VA 24061, USA; ^2^Toshiba Medical Research Institute USA, Vernon Hills, IL 60061, USA

## Abstract

Although x-ray imaging is widely used in biomedical applications, biological soft tissues have small density changes, leading to low contrast resolution for attenuation-based x-ray imaging. Over the past years, x-ray small-angle scattering was studied as a new contrast mechanism to enhance subtle structural variation within the soft tissue. In this paper, we present a detection method to extract this type of x-ray scattering data, which are also referred to as dark-field signals. The key idea is to acquire an x-ray projection multiple times with varying collimation before an x-ray detector array. The projection data acquired with a collimator of a sufficiently high collimation aspect ratio contain mainly the primary beam with little scattering, while the data acquired with an appropriately reduced collimation aspect ratio include both the primary beam and small-angle scattering signals. Then, analysis of these corresponding datasets will produce desirable dark-field signals; for example, via digitally subtraction. In the numerical experiments, the feasibility of our dark-field detection technology is demonstrated in Monte Carlo simulation. The results show that the acquired dark field signals can clearly reveal the structural information of tissues in terms of Rayleigh scattering characteristics.

## 1. Introduction 

Since its invention in 1973 [1], X-ray computed tomography (CT) has revolutionized medical imaging and become a cornerstone of modern radiology. Improving resolution and reducing dose are two critical factors in biomedical applications and remain the focuses of CT research. With the emergence of multislice spiral CT in 1998, cone-beam scanning is recognized as a major mode for medical CT and widely used in numerous diagnostic and therapeutic procedures. Moreover, the rapid development of small animal models, especially those with genetically engineered mice [2–4], has generated the need for preclinical imaging, reaching image resolution in the micron range. These scanners, while producing high spatial resolution images, do not allow high contrast and low dose imaging in either patients or animal models. For example, many normal and diseased tissues such as cancers display poor image contrast in current X-ray images as they have very similar attenuation characteristics.

X-ray mammography is currently the most prevalent imaging modality for screening and diagnosis of breast cancers. The use of mammography results in a 25%–30% decreased mortality rate in screened women [5]. However, a multi-institutional trial funded by the American College of Radiology Imaging Network (ACRIN) suggested that ~30% of cancers were not detected by screening mammography, and 70%–90% of biopsies performed based on suspicious mammograms were negative [6, 7]. Some false negative and false positive diagnoses often led to missed cancers and inappropriate biopsies. The key factor that limits the success rate is the poor contrast between healthy and diseased tissues in the mammogram. Although X-ray CT of the breast can potentially improve diagnostic accuracy over mammography [8–10], the state-of-the-art breast CT scanner is still based on the attenuation mechanism. As a result, the use of breast CT requires an intravenous contrast medium and a high radiation dose, since elemental composition is almost uniform with little density variation in breast tissues. Still, it is rather difficult for breast CT to discern early-stage breast cancers [11–13].

Driven by major practical needs for better X-ray imaging, exploration into contrast mechanisms other than attenuation has been active for decades, especially in terms of small-angle scattering (essentially, Rayleigh scattering) [14–17] and refraction of X-rays [17–19], which are also known as dark-field and phase-contrast imaging, respectively. Up to now, X-ray Rayleigh scattering-based imaging has been limited to in vitro studies, incapable of volumetric cone-beam scanning, lack of rigorous reconstruction theory, and made little progress into clinical practice. Since 2006, grating-based X-ray dark-field and phase-contrast tomography is being developed using a hospital-grade X-ray tube, instead of a synchrotron facility or microfocus tube [20]. This technology utilizes the optical interference principles to yield high-quality dark-field images. The boundaries and interfaces in the biological tissues produce strong signals in dark-field images, indicating detailed structural contours. Moreover, dark-field images have greater signal-to-noise ratios in soft tissues than bright-field counterparts acquired with the same incident X-ray dose. However, the major problems with this grating-based approach are small sample size, long imaging time, and high fabrication cost. 

In this paper, we present a varying collimation scheme to extract dark-field signals. The key idea is to acquire X-ray projection data multiple times with varying collimation. In the following section, we describe the principles of our dark-field detection method. In the third section, we present our initial simulation results to show the feasibility of our methodology. In the last section, we discuss relevant issues.

## 2. Methodology

The trick of the varying collimation scheme is to acquire each X-ray sum twice with different collimation aspect ratios. The projection data acquired with a collimator of a sufficiently large aspect ratio contain little scattering, while the corresponding data acquired with the collimator of an appropriately reduced aspect ratio include both small-angle scattering and primary signals. Then, analysis of these paired datasets will produce desirable dark-field signals, in addition to traditional transmission measurement.

Conventionally, an antiscattering grid is coupled with an area detector to eliminate X-ray scattering photons. The intensity of scattered radiation into a detector cell is determined by the height of the antiscattering grid. The lower the height of the antiscattering grid is, the more the scattered photons enter the detector cell. The height of the antiscattering grid is typically selected to reject scattered photons as much as feasible subject to the cost associated with the fabrication process. In our approach, depending on a specific imaging application we can choose a height of the antiscattering grid appropriately so that only the primary and small-angle scattering signals are intercepted. The resultant projection is denoted as PS. Then, we can increase the height of the antiscattering grid significantly so that small-angle scattering signals are also rejected to acquire essentially only the transmission data. The corresponding projection is denoted as PT. Hence, the difference between PS and PT should be closely correlated to the desirable small-angle scattering signals.

**Figure 1:**
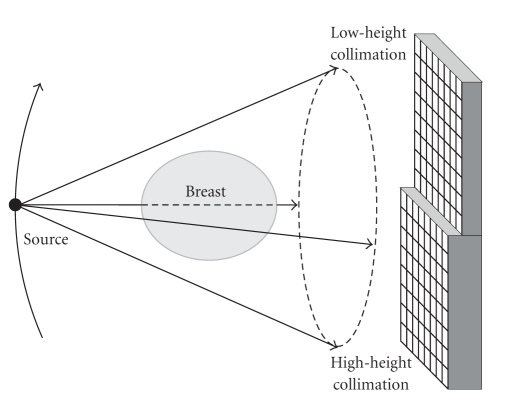
Single pass cone-beam circular scan with a dual collimation detector array for both dark-field tomography and transmission X-ray CT.

It is underlined that our varying collimation approach does not necessarily require two pass scans with different collimation aspect ratios. For example, as shown in Figure 1, for a circular cone-beam full-scan we can use a dual-height collimator in front of a 2D detector array. In this setting, on the mid-plane each pixel on a given line is irradiated by two and only two X-rays along that same line but with two different collimation heights in the opposite directions respectively, sufficient for extraction of dark-field signals. 

## 3. Numerical Simulation

The small-angel and large-angle scattering signals come essentially from the coherent scattering (Rayleigh scattering) and incoherent scattering (Compton scattering) mechanisms, respectively. Compton scattering describes the interaction of a photon with an electron in an outer shell of an atom. A fraction of the X-ray energy is transferred to the electron. While the electron is ejected, the X-ray photon is deflected from its original path. The probability for an incoming photon with energy *E*_*γ*_  being scattered in an direction *θ*  can be described by the Klein-Nishina formula [21]

(1) $$p_{c}\left(\theta \right)=\frac{r_{e}^{2}}{2}\left(1+{\cos\;}^{2}\theta +\frac{\alpha^{2}{\left(1-\cos\;\theta \right)}^{2}}{1+\alpha \left(1-\cos\;\theta \right)}\right)\frac{1}{{\left(1+\alpha \left(1-\cos\;\theta \right)\right)}^{2}},$$

where *α* = *E*_*γ*_/*m*_*e*_ *c* ^2^, *m*_*e*_ is the electron mass, *c* the speed of light, and *r*_*e*_ the classical radius of electron. 

Rayleigh scattering represents non-ionizing interactions between X-rays and matters. It is an elastic scattering process. The scattered photons have the same energy as the incident photons. The differential cross-section of Rayleigh scattering is as follows [22]:

(2) $$p_{r}\left(\theta \right)=\frac{r_{e}^{2}}{2}\left(1+{\cos\;}^{2}\theta \right)F^{2}\left(\theta ,E_{\gamma },Z\right),$$

where *F*(*θ*, *E*_*γ*_, *Z*) is the atomic form factor. Since the form factor is highly complex, most X-ray Monte Carlo simulators use a database to store the form factor data. It is desirable and feasible to use a simple function to approximate the form factor as *F*(*θ*, *E*_*γ*_, *Z*) = *c*_1_ *θ* ^*l*^ *e* ^−*c*_2_*θ*^. 

The combined differential cross-section per atom can be expressed as

(3) $$p^{\prime }\left(\theta \right)=p_{r}\left(\theta \right)+N_{e}p_{c}\left(\theta \right),$$

where *N*_*e*_ is the number of free electrons in the atom. 

Let us define the scattering-induced linear attenuation coefficient as

(4) $$\mu_{s}=2\pi n_{s}\int_{0}^{\pi }d\theta p\prime \left(\theta \right)=n_{s}\sigma_{s},$$

where *n*_*s*_ indicates the number density of scatter atoms, and *σ*_*s*_ = 2*π*∫_0_^*π*^ *d* *θ* *p*′(*θ*) the total scatter cross-section. The combined probability of Rayleigh and Compton scattering becomes

(5) $$p\left(\theta \right)=\frac{1}{\sigma_{s}}p^{\prime }\left(\theta \right).$$

The total Rayleigh probability can be defined as *β* = *σ*_*r*_/*σ*_*s*_, where *σ*_*r*_ = 2*π*∫_0_^*π*^ *d* *θ* *p*_*r*_(*θ*). 

### 3.1. Single Scattering Method

**Figure 2:**
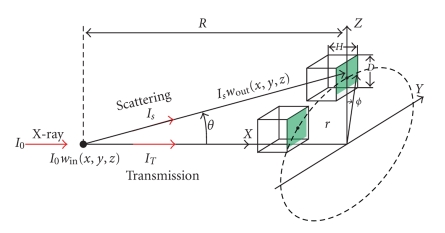
Geometry for detection of X-ray scattering signals.

For a proof of concept, first we used a single scattering model in 3D to demonstrate our varying collimation technology. Although the single scattering model represents the simplest X-ray scattering scenario, it is sufficient as the initial pilot study. As shown in Figure 2, the X-ray scattering intensity into a detector cell can be expressed as:

(6) $$\begin{array}{cc} f\left(H\right) & =\frac{1}{2\pi }\int dz\int dy\int dxI_{0}w_{\text{in}}\left(x,y,z\right)w_{\text{out}}\left(x,y,z\right)\mu_{s}\left(x,y,z\right)\\ & \;\times \int_{\phi_{1}}^{\phi_{2}}d\phi \int_{\theta_{1}}^{\theta_{2}}d\theta \;p\left(\theta \right), \end{array}$$

where (1/2*π*)∫_*ϕ*_1__^*ϕ*_2_^ *d* *ϕ*∫_*θ*_1__^*θ*_2_^ *d* *θ* *p*(*θ*) is the probability that a scattered photon hits a target detector pixel, *I*_0_ the source intensity, *w*_in_(*x*, *y*, *z*) the source attenuation factor, *w*_out_(*x*, *y*, *z*) the scattering signal attenuation factor which also depends on the location of the detector cell, and *μ*_*s*_(*x*, *y*, *z*) the scattering coefficient. In Figure 2, *p*(*θ*) represents a symmetry distribution around the incoming direction of a photon. Hence, when computing the differential solid angle extended by an arbitrary detector cell we can always rotate the detector cell such that its center is on the *X-Z* plane as an approximation (which is equivalent to assuming the rotational invariance of each collimator unit). Generally speaking, we can use the following limits to compute this probability approximately:

(7) $$\begin{array}{c} \;\theta_{1}=\arctan\left(\frac{r-D/2}{R-H}\right),\;\;\theta_{2}=\arctan\left(\frac{r+D/2}{R}\right),\\ \phi_{1}=-\arctan\left(\frac{D/2}{r}\right),\;\;\phi_{2}=\arctan\left(\frac{D/2}{r}\right), \end{array}$$

where *H* is the grid height, *D* the aperture of the detector cell, and *R* the distance from a scattering location to the detector cell. Note that the scattering behavior (1/2*π*)∫_*ϕ*_1__^*ϕ*_2_^ *d* *ϕ*∫_*θ*_1__^*θ*_2_^ *d* *θ* *p*(*θ*) can be analytically computed, numerically estimated, or statistically simulated.

### 3.2. Monte Carlo Simulation Method

Recently, we developed a tetrahedron-based inhomogeneous Monte Carlo optical simulator (TIM-OS) for optical light propagation in complex biological tissues [23]. TIM-OS can move particles efficiently in a complex geo*metr*y represented by a tetrahedral mesh. Since we achieved a great speedup in optical simulation, we migrated the TIM-OS framework for X-ray simulation as well. 

X-ray and matter interaction is very complex in general. In this prototype MC X-ray simulator, a simplified X-ray-matter interaction model was considered to cover the three major components in dark-field imaging: Photoelectric effect (absorption), Rayleigh scattering, and Compton scattering. We used three parameters to describe the X-ray-matter interaction: absorption coefficient (*μ*_*t*_), scattering coefficient (*μ*_*s*_), and Rayleigh percentage (*β*). The definition of absorption coefficient is the probability of X-ray absorption per unit path length. The scattering coefficient is the probability of an X-ray photon involved in scattering (Rayleigh or Compton scattering) per unit path length. Rayleigh percentage determines the likelihood of Rayleigh scattering in a scattering event. 

After an X-ray photon is launched, a routine will be followed to find out the entering point of the photon into the phantom. While the photon is in the phantom, a step size will be generated based on the local absorption and scattering coefficients as *s* = −ln (*ξ*)/(*μ*_*t*_ + *μ*_*s*_), where *ξ* is a uniform random number from (0,1] [24]. If this photon needs to go across several different regions, the total step size *s* = ∑_*i*_ *s*_*i*_ is governing by the following equation: ∑_*i*_(*μ*_*t*_*i*_ + *μ*_*s*_*i*_)*s*_*i*_ = −ln (*ξ*). After the photon moves the free fly step, the photon will be absorbed or scattered based on the ratio of the absorption coefficient and scattering coefficient. If the photon is absorbed, the program will launch a new photon; otherwise, the photon is scattered. According to *β*, the photon scattering will be governed by either the Rayleigh or Compton mechanism. Then, the scattering angle will be found according to the corresponding form factor. Then, the photon will be assigned another step size based on the current local parameters. These steps will be repeated until the photon moves out of the phantom. 

Voxel-based and surface-based schemes are two popular techniques employed in X-ray simulation to deal with a complex geometry [25, 26]. The surface-based scheme uses a triangle mesh to represent the interface between two regions and the surface. In this case, a simulation program needs to determine whether the involved photon moment will hit a triangle for each step. Given a complex geometry, the computation of photon-triangle interaction could be very slow. The voxel-based scheme directly uses a CT reconstruction volume to represent geometry. This may introduce a huge computational overhead when a high-resolution volumetric image is used. The key idea underlying our tetrahedron-based scheme is that by modeling an object as a tetrahedron-based finite element mesh, TIM-OS can specify the photon-triangle interaction rapidly and recursively. In other words, since a photon starts its movement inside a tetrahedron, the ray-triangle interaction would only happen with one of the four triangles of that tetrahedron, reducing the searching space significantly [23].

### 3.3. Simulation Result

**Figure 3:**
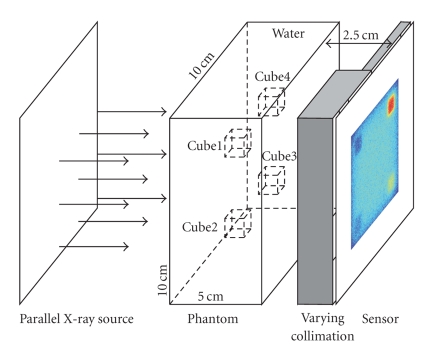
Simulation setting for imaging based on small-angle scattering.

In this section, we will use Monte Carlo simulation to verify the varying collimation scheme and compare the single scatter method with Monte Carlo simulation result. Figure 3 illustrates the simulation setting. A 10 × 10 × 5 cm^3^ phantom with four 1 cm cubic subregions was used in this study. The phantom material was set to water. Furthermore, the four cubic subregions were made of the same attenuation coefficient as water but with different scattering behaviors. Based on [27], at 50 KeV water's total attenuation coefficient is 0.21(cm^−1^). In the attenuation coefficient, the absorption is about 13.3% (0.028 cm^−1^), the Rayleigh scattering (*μ*_*s*_*r*_) is 6.7% (0.014 cm^−1^), and Compton scattering (*μ*_*s*_*a*_) is 80% (0.168 cm^−1^). Two of the cubes had lower Rayleigh scattering coefficients, and the other two had higher Rayleigh scattering coefficients. Table 1 lists the X-ray absorption and scattering coefficients at 50 KeV of the phantom components. 

**Figure 4:**
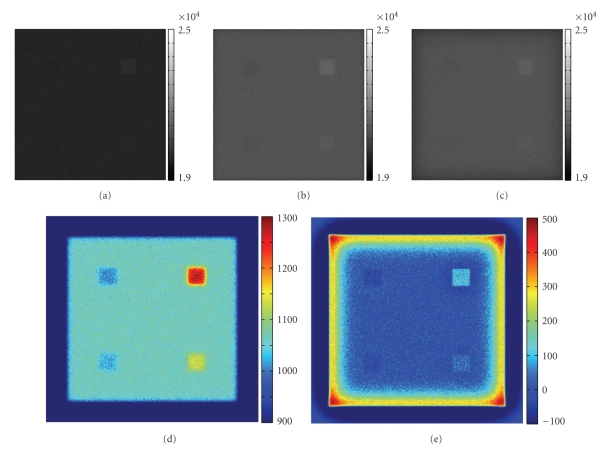
Numerical results on X-ray small-angle scattering imaging. (a) An image collected with a high collimation aspect and a small object-detector distance (2.5 cm), (b) an image with a low collimation aspect and a small object-detector distance (2.5 cm), (c) an image with a low collimation aspect and a large object-detector distance (17.5 cm), (d) the difference between (a) and (b), and (e) the difference between (b) and (c).

The pixel size of the detector was set to 0.2 × 0.2 mm^2^ and there were a total of 600 × 600 pixels to cover the whole phantom area. The distance between the phantom and the detector plane was 2.5 cm. In the simulation, we adjusted the form factor (*θ* ^*l*^ *e* ^−*c*_2_*θ*^) for Rayleigh scattering such that the average Rayleigh scattering angle was 4.3°. In each run, TIM-OS traced a total of 2 × 10^10^ X-ray photons. Figures 4(a) and 4(b) presents two images obtained with the varying collimation method: an image obtained with a collimator of a high collimation aspect ratio 50 (IH), and a counterpart with a low collimation aspect ratio 10 (IL). By subtracting IH from IL, we estimated the Rayleigh scattering image (Figure 4(c)). The varying collimation scheme correctly extracted the small angle scattering signals, and the signal intensities reflected the relative Rayleigh scattering percentages. Hence, it is indeed feasible to reconstruct not only attenuation but also Rayleigh scattering parameters based on the varying collimation scheme. Note that we can also extract the small angle scattering information by capturing two images at different object-detector distances without changing the detector collimator physically. By subtracting the longer distance image from the shorter distance image, we can digitally extract the small-angle scattering information. Figure 4(c) is an image captured with a longer distance (17.5 cm) than Figure 4(b) (2.5 cm) given the same collimation ratio (10). Figure 4(e) shows the difference between these two images. 

**Figure 5:**
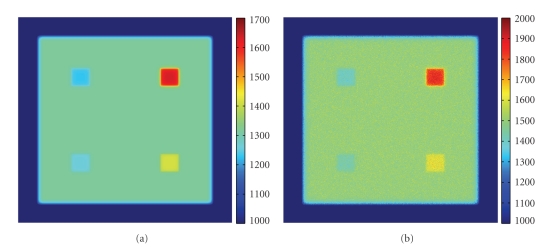
Numerical results on X-ray single scattering imaging. (a) A single scattering image from (6)-(7), and (b) a pure small angle scattering image from the Monte Carlo simulation.

While Monte Carlo simulation provides the gold standard for small scattering imaging, the single scattering method provides a faster way to estimate the small scattering signal. Thus, we used (6) to predict the single scattering image for the phantom in Figure 3 by assuming a low collimation ratio 10 and short detector-object distance 2.5 cm. Here the computation of (1/2*π*)∫_*ϕ*_1__^*ϕ*_2_^ *d* *ϕ*∫_*θ*_1__^*θ*_2_^ *d* *θ* *p*(*θ*) was completed in a Monte Carlo simulation in advance for 250 (=5 cm/0.02 cm) different depths and 5 different materials. Figure 5 shows the numerical result according to the single scattering model (6)-(7) and the Monte Carlo simulated small-angle scattering image. The Monte Carlo simulation took multiple scattering signals into account. Quantitatively, the Monte Carlo simulated small scattering image is about 10% higher than the single scattering image, which shows the validity and utility of the single scattering model in this type of applications. 

## 4. Discussions and Conclusion

Actually, varying the collimator height gives us a new dimension to analyze scattering signals. We may use two or more collimator heights, depending on specific application requirements. Also, it is possible to vary the X-ray tube voltage for more information. The resultant projection data are not exact line integrals. Accurate reconstruction algorithms should reflect the transport process, somehow between classic X-ray CT and diffuse optical tomography (DOT). We are actively working in this direction.

In conclusion, we have proposed a varying collimation methodology for extraction of the dark-field signal for dark-field tomography. Our method is advantageous in several aspects. Practically, it can be implemented by modifying the existing collimation technology slightly. Technically, it allows volumetric scanning such as in circular and spiral cone-beam geometries. Potentially, it may be extended to probe other X-ray interactions with materials. The proposed approach has an implication for a wide range of applications including medical imaging, security screening, industrial nondestructive testing, and so on.

## Figures and Tables

**Table 1:** X-ray absorption and scattering coefficients at 50 KeV of the phantom components.

Material	Absorption Coefficient (cm^−1^)	Scattering coefficients (*μ*_s_ = *μ*_s_r_ +*μ*_s_c_ ) (cm^−1^)		
		*μ*_s_r_	*μ*_s_c_	*μ*_s_r_/(*μ*_s_r_ + *μ*_s_c_)
Water	0.028	0.0140	0.1680	0.0769
Cube 1	0.028	0.0035	0.1785	0.0193
Cube 2	0.028	0.0070	0.1750	0.0385
Cube 3	0.028	0.0260	0.1560	0.1429
Cube 4	0.028	0.0520	0.1300	0.2858
